# Long-term physical workload in middle age and disability pension in men and women: a follow-up study of Swedish cohorts

**DOI:** 10.1007/s00420-016-1156-0

**Published:** 2016-07-30

**Authors:** Katarina Kjellberg, Andreas Lundin, Daniel Falkstedt, Peter Allebeck, Tomas Hemmingsson

**Affiliations:** 1 Institute of Environmental Medicine, Karolinska Institutet, Solnavägen 4, 10th Floor, 113 65 Stockholm, Sweden; 2 Centre for Occupational and Environmental Medicine, Stockholm County Council, Stockholm, Sweden; 3 Department of Public Health Sciences, Karolinska Institutet, Stockholm, Sweden; 4 Centre for Social Research on Alcohol and Drugs, Stockholm University, Stockholm, Sweden

**Keywords:** Cohort study, Education, IQ, Job control, Job exposure matrix, Musculoskeletal disorder

## Abstract

**Purpose:**

The study investigates the association between level of long-term physical workload in middle age and disability pension (DP) before 61 years of age with adjustments made for early life factors, level of education, and psychosocial working conditions. Associations with DP overall, DP due to musculoskeletal disorders and DP due to psychiatric disorders were examined.

**Methods:**

The study is based on cohorts of 21,809 Swedish men and women born in 1948 and 1953, with data on physical workload estimated with a job exposure matrix based on occupational titles in 1985 and 1990 and follow-up data on diagnosis-specific DP in the years 1991–2009. Data on paternal education and intelligence were collected in primary school. Data on level of education were taken from administrative records. Data on psychosocial working conditions were estimated with a job exposure matrix based on occupational titles in 1990.

**Results:**

Long-term exposure to high physical workload measured 5 years apart at around age 40 was strongly associated with DP due to musculoskeletal disorders up to the age of 61 among both men (HR 5.44, 95 % CI 3.35–8.84) and women (HR 3.82, CI 95 % 2.88–5.08). For women, the association between high physical load and overall DP was also significantly increased (HR 2.33, CI 95 % 1.92–2.82). The increased risks remained but were clearly attenuated after adjustments for fathers’ education, IQ in childhood, achieved education and level of control at work.

**Conclusions:**

Exposure to high physical workload is associated with long-term risk of DP due to musculoskeletal disorders, even though adjustments for early life factors, level of education and psychosocial working conditions clearly attenuated the risks.

## Introduction

**Fig. 1:**
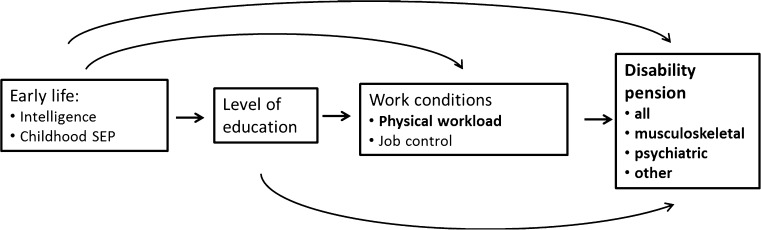
Potential pathways leading to a disability pension explored in this study

Associations between occupational exposures for high physical load, for example heavy manual handling and working in awkward work postures, and musculoskeletal disorders are well documented (da Costa and Vieira 2010; Punnett and Wegman 2004), even though there is still a debate concerning the work-relatedness of musculoskeletal disorders (Punnett 2014; Punnett and Wegman 2004). High physical workload has also been associated with early exits from the labor market through disability pensions (DP) in a number of studies, for example (Friis et al. 2008; Jarvholm et al. 2014; Karkkainen et al. 2013; Karpansalo et al. 2002; Labriola et al. 2009b; Lahelma et al. 2012). In studies of DP due to specific diagnosis groups, strong effects of physical workload on DP due to musculoskeletal disorders have been indicated (Karkkainen et al. 2013; Karpansalo et al. 2003; Lahelma et al. 2012), but no effect on DP due to mental disorders (Karpansalo et al. 2002; Lahelma et al. 2012) or cardiovascular disease (Karpansalo et al. 2002). However, the association between high physical workload and DP may be confounded by early life risk factors, level of education and psychosocial working conditions (Fig. 1).

In Sweden, disability pension benefits are available through the social insurance system for adults up to the regular retirement age of 65. Decisions of DP benefits are based on a permanently reduced work ability by at least 25 % due to a disease or other physical or mental functional impairment (SFS 2010). Depending on the level of reduced work ability, the allowance of DP can be full, three-quarter, half or a quarter. The most common diagnoses for receiving DP in Sweden are musculoskeletal and psychiatric diagnoses (SSIA 2015). In most cases, a DP implies a permanent exit from the labor market. Causes of the large amounts of early exits from working life through disability pensions, for example in OECD countries (OECD 2010), need to be identified. The large differences in the risk of receiving a DP shown between occupational groups (Haukenes et al. 2011; Krokstad et al. 2002; Lahelma et al. 2012; Leinonen et al. 2011), and occupations (Jarvholm et al. 2014) indicate that working conditions as risk factors needs to be examined.

In addition to physical working conditions, adverse psychosocial working conditions have been reported as determinants of DP, for example low job satisfaction (Labriola et al. 2009a), high job strain (Laine et al. 2009), and low job control (Krokstad et al. 2002; Lahelma et al. 2012). Jobs involving heavy manual works tasks are often characterized by low control of the worker (Kausto et al. 2011; MacDonald et al. 2001; Schrijvers et al. 1998). Only a few studies on the association between high physical workload and DP have been found adjusting for psychosocial working conditions in the analyses (Friis et al. 2008; Krokstad et al. 2002; Labriola et al. 2009b; Lahelma et al. 2012); all showing that the associations remain. Lahelma et al. (2012) made a comprehensive examination of associations between a wide range of working conditions and DP. They found that, among the psychosocial factors, low job control was the most consistent risk factor.

Risk factors for DP have also been identified from earlier life before the entrance to working life. There is a possibility that early factors act as selective forces into working life, and may also confound the association between high physical workload during working life and DP (Fig. 1). Such a factor is low educational attainment, which has been strongly associated with DP in previous studies (Bruusgaard et al. 2010; Gravseth et al. 2007; Johansson et al. 2012; Krokstad et al. 2002; Nilsen et al. 2012), particularly with DP due to musculoskeletal disorders (Bruusgaard et al. 2010; Falkstedt et al. 2014). Youths with low educational level are probably selected into low-skilled jobs often characterized by manual work tasks and high physical load (Schrijvers et al. 1998; Warren et al. 2004). It has been shown that adverse working conditions explain parts of the association between low education and DP (Falkstedt et al. 2014; Johansson et al. 2012; Krokstad et al. 2002; Nilsen et al. 2012).

Factors measured even earlier in life, such as birth weight (Gravseth et al. 2007), intelligence (Lundin et al. 2015; Sorberg et al. 2013; Upmark et al. 2001), socioeconomic position (SEP) in childhood (Upmark et al. 2001; Upmark and Thundal 2002), parental disability (Gravseth et al. 2007) and unfavorable childhood experiences (Upmark and Thundal 2002), have also been associated with DP. Some of these factors have been shown to contribute to the association between educational level and DP (Falkstedt et al. 2014; Johansson et al. 2012; Upmark et al. 2001); for example SEP in childhood (Falkstedt et al. 2014) and intelligence (Johansson et al. 2012). A higher rate of DP among persons who have been exposed to high physical load in working life may, apart from being explained by the well-documented harmful effects of mechanical load to the musculoskeletal system, to some extent also be a result of selection forces from early life into education and jobs. In some of the previous studies on physical workload and DP, confounding by education or SEP has been controlled for and associations have remained (Karpansalo et al. 2002; Krokstad et al. 2002; Lahelma et al. 2012). However, most studies have not controlled for potential confounding due to early life factors.

In summary, the association between high physical workload during working life and DP may be confounded by early life factors, level of education and psychosocial working conditions (Fig. 1). These factors may be unevenly distributed among employees with different levels of physical workload. For example, in occupational groups with high physical load, i.e. manual work, a higher prevalence of low educational level and low job control could be expected. Early life factors, such as SEP during childhood and intelligence, could influence the choice of education and occupation, as well as being risk factors in themselves for DP. To our knowledge, no study exists where the association between high physical workload and DP has been adjusted for early life factors, educational level, and psychosocial working conditions together. Also, in most studies on high physical workload and DP, information on workload has been gathered only from one occasion, for example (Friis et al. 2008; Karpansalo et al. 2002; Lahelma et al. 2012), potentially diluting the associations with temporary exposure levels.

The aim of this study was to investigate the association between level of long-term physical workload in middle age and DP before 61 years of age. Adjustments were made for early life factors, educational level, and psychosocial working conditions. We studied associations both with DP overall and with DP due to musculoskeletal disorders or psychiatric disorders, as previous studies suggest that determinants may differ between DP according to diagnosis group (Karpansalo et al. 2003; Lahelma et al. 2012). Unlike most previous studies, the measure of high physical workload was based on long-term exposure, based on 5 years of stable exposure, and not self-reported, but based on a job exposure matrix (JEM).

## Methods

### Participants and study design

The study is based on two cohorts of Swedish schoolchildren born in 1948 (*n* = 11,928) and 1953 (*n* = 9881) which were followed up for educational achievement as part of the project “Evaluation through Follow-up” (Harnqvist 1998). The cohorts were derived from 10 % samples of all Swedish schoolchildren in grade 6 (approximately at age 13) identified by selecting pupils born on the 5th, 15th, and 25th of each month of the year. The pupils were surveyed in 1961 and 1966, respectively, by intelligence quotient (IQ) tests and questionnaires administered in school (Table 1). The participation rate was 98 and 93 %, respectively.

**Table 1:** Timing of data collection for two cohorts of schoolchildren

Data	Years	Ages	Register/database
Birth year	1948/1953	0	The school survey
Paternal education	1961/1966	13	The school survey
Intelligence	1961/1966	13	IQ tests from the school survey
Level of education	1986	38/33	Administrative school records
Physical workload	1985	37/32	National Population and Housing Censuses
	1990	42/37	
Job control	1990	42/37	National Population and Housing Censuses
Disability pension	1991–2009	43/38–61/56	Swedish Social Insurance Agency’s database

The two cohorts were analyzed together in order to increase the power of the study and consisted of 21,809 individuals, 11,100 men and 10,709 women. Data were linked to individual information from registers and censuses on own education, occupation (1985 and 1990) and date and cause of DP during 1991–2009 (Table 1).

Ethical approval was obtained from the ethics committee at the Karolinska Institute, Stockholm.

### Physical workload (exposure)

Physical workload was estimated with a JEM based on occupational histories of respondents age 25–74 in the Swedish Annual Survey of Living Conditions 1977 and 1979–1981 (Östlin 1989b). An index was created based on questions concerning eight different physical risk factors: heavy lifts daily, repetitive and one-sided work movements, awkward work postures, heavy shaking or vibrations, daily perspiration from physical exertion, contact with dirt, deafening noise, and risk for exposure to accidents. Mean levels, by sex, of the index were computed for more than 300 occupations (coded according to the 1973 version of Nordic Occupational Classification system, NYK, which follows the three digit International Classification ISCO), and the mean levels were grouped into quartiles; high, medium–high, medium–low or low physical workload. Through NYK codes, we assigned these four categories of physical workload to the occupation stated in the National Population and Housing Censuses 1985 and 1990 (Table 1). For both men and women, the mean index for the NYK group has been shown to have a satisfactory correlation with individuals’ own index (Spearman correlation for males *r* = 0.52, females *r* = 0.51) (Lundin et al. 2015). A category of stable exposure to physical workload was created where only individuals who had been assigned the same category of physical workload in 1985 and 1990 were included.

### Disability pension (outcome)

Information on disability pension is registered yearly in the Swedish Social Insurance Agency’s register. To receive a DP in Sweden, the work ability must be permanently reduced with at least 25 % due to a disease or other reduction of the physical or mental capacity (SFS 2010). Causes of DP were grouped into musculoskeletal (ICD-9: 710–739, ICD-10 M01–M99), psychiatric (ICD-9: 290–311, ICD-10 F00–F99) and “other” causes, i.e. the remaining ICD-codes.

The outcome on DP was collected between 1991 and 2009, when the subjects in the two cohorts were 43 and 38 years, respectively, or older. Data were obtained on time to first DP episode (both full and partial DP).

### Covariates

#### Intelligence

IQ tests (antonyms, metal folding, and number series tests) were performed at the age of 13. The three tests were summarized into a score according to standardized procedures (Harnqvist 1968a, b). The score was transformed into a normalized standard nine point scale (Stanine) with scores ranging from 1 (low) to 9 (high), with a mean of 5, as a measure of general intelligence.

#### Paternal education

The highest level of education of the father (or other male caretaker) was reported in a questionnaire at the age of 13 with four predefined categories: primary school (compulsory 6 years), lower secondary school (9 years), upper secondary school (12 years) and higher education (more than 12 years). We dichotomized the data into primary school (low education) and more (not low) because of a highly skewed distribution.

#### Job control

Job control was estimated with a JEM based on occupational histories of respondents in the Swedish Work Environment Surveys 1989–1997 (Fredlund et al. 2000). The surveys included questions on different aspects of the psychosocial work environment, e.g. job control. Job control, or decision latitude, is a combined measure based on two scales measuring decision authority (four items) and skill discretion (three items). Mean levels of the job control index, by sex and age, were calculated for 320 occupations (coded according to the 1983 version of Nordic Occupational Classification system). The correlation between the JEM control mean and individuals’ control index has been shown to be satisfactory (Spearman correlation for blue collar and white collar workers age 30–44 ranged *r* = 0.39–0.44 in men and *r* = 0.41–0.45 in women) (Fredlund et al. 2000). These mean levels were assigned to our study participants’ NYK codes occupation stated in the National Population and Housing Censuses 1990 and were grouped into quartiles labeled high, medium–high, medium–low or low control at work.

#### Level of education

Data on level of education were taken from administrative school records in 1986 and registered according to the Swedish version of the International Standard Classification of Education (ISCED): (1) primary and lower secondary, <9 years; (2) primary and lower secondary, 9 years; (3) secondary; (4) upper secondary; (5) postsecondary, 2 years or less; (6) postsecondary, 3–4 years; and (7) postgraduate education. We combined categories 1 and 2 (≤9 years of schooling), and 6–7 (≥15 years of schooling) leaving five groups based on number of years of education: ≤9, 10–11, 12–13, 14, and ≥15 years.

### Data analysis

The study population consisted of those 21,809 persons (11,100 men and 10,709 women) who were surveyed in the 6th grade in school. Among these, 458 persons had died before 1991, leaving 21,351 individuals. Of these, 626 persons had been granted a disability pension before 1991 and were therefore excluded, leaving 20,725 individuals. Another 3070 persons for whom we lacked occupational information, needed for occupational exposure classification, were also excluded. The lack of occupational information was either due to that individuals had not participated in the National Population and Housing Censuses or that reported occupations could not be classified by NYK. In the remaining population of 17,655 persons, 4426 persons did not have a stable exposure to physical workload in 1985 and 1990 and were therefore excluded. Additionally 1304 persons lacked information on either paternal education, own education or IQ test results. Thus, the population followed from 1991 to 2009 consisted of 11,925 persons (6328 men and 5597 women) (Table 2).

The proportion of men and women receiving a DP (overall and in different diagnosis groups) were calculated for each level of physical workload. We also calculated the prevalence of covariates across levels of physical workload.

The association between level of stable physical workload at 1985–1990 and incident cases of disability pension was estimated by means of Cox proportional-hazard models, yielding hazard ratios (HR) with 95 % confidence intervals (CI). The regressions were computed in the SAS software package using the PHREG procedure (SAS 9.1, SAS Institute Inc., Cary, NC, USA). Person-years were counted from January 1, 1991, until the date of receiving first-time DP, date of death, date of emigration or until end of follow-up on July 1, 2009. Non-proportionality of hazards (confounding by time) was investigated through examining the hazard ratios in smaller time bands of the follow-up period. All regressions were adjusted for birth cohort (year of birth 1948 or 1953). In the analyses, we first investigated the association between level of physical workload and disability pension without covariates. Stratified analyses were done for women and men. Adjustments were then made for potential confounding factors from early life (age 13), level of education collected from 1986, and psychosocial working conditions collected from 1990.

## Results

Of the 11,925 individuals with a stable exposure to physical workload in 1985 and 1990, there were 1626 incident cases of disability pensions; 620 in men and 1006 in women, during the 19-year follow-up. Table 2 shows the distribution of overall DP and DP in different diagnosis groups, across physical workload categories. There was a considerably higher proportion of women receiving a DP compared with men across all physical workload levels and regardless of diagnosis group.

**Table 2:** Study population distributed by level of physical workload in middle age and prevalence of disability pension in each level of physical workload among middle-aged men and women

Full cohorts	No of persons with information on physical workload 1985–1990 and included in the analyses		Disability pensioners 1991–2009							
	Physical workload		Total		Musculoskeletal		Psychiatric		Other	
		*n*	*n*	%	*n*	%	*n*	%	*n*	%
Men	High	1808	229	12.7	115	6.4	30	1.7	84	4.7
	Medium–high	1134	150	13.2	52	4.6	29	2.6	69	6.1
	Medium–low	1896	142	7.5	38	2.0	36	1.9	68	3.6
	Low	1490	99	6.6	19	1.3	35	2.4	45	3.0
All										
11,100		6328	620	9.8	224	3.5	130	2.1	266	4.2
Women	High	482	147	30.3	82	17.0	23	4.8	42	8.7
	Medium–high	1163	261	22.4	132	11.4	47	4.0	82	7.1
	Medium–low	1666	257	15.4	104	6.2	73	4.4	80	4.8
	Low	2286	341	14.9	115	5.0	104	4.6	122	5.3
All										
10,709		5597	1006	18.0	433	7.7	247	4.4	326	5.8

The level of physical workload was strongly correlated to early life factors, educational level and coincident job control among both men and women (Table 3). Women and men with a high and medium–high physical workload at middle age had more often had a low SEP during childhood (based on father’s education), a low IQ at the age of 13, a low educational level and a low job control at middle age compared to those with a low and medium–low physical workload. For women, also those with a medium–low physical load had more often a low control at work compared to those with a low physical load. Men with a medium–high physical workload had the highest proportion of low control at work among the men, higher than both those with high physical workload and those with low and medium–low load. At all physical workload levels, women had higher proportions of low job control than men. The largest difference between men and women was seen in the highest workload category where 88 % of the women compared to 26 % of the men had low job control.

**Table 3:** Prevalence of covariates in different workload categories

	Overall		Physical workload 1985–1990							
			Low		Medium–low		Medium–high		High	
	*n*	%	*n*	%	*n*	%	*n*	%	*n*	%
Men	6328		1490		1896		1134		1808	
Low paternal education^a^	5169	81.7	1031	69.2	1432	75.5	1029	90.7	1677	92.8
Low intellectual performance^b^	1302	20.6	167	11.2	230	12.1	352	31.0	553	30.6
Low education^c^	1993	31.5	209	14.0	281	14.8	553	48.8	950	52.5
Low job control	1197	18.9	21	1.41	86	4.54	621	54.8	469	25.9
Women	5597		2286		1666		1163		482	
Low paternal education^a^	4543	81.2	1739	76.1	1309	78.6	1052	90.5	443	91.9
Low intellectual performance^b^	1221	21.8	314	13.7	287	17.2	400	34.4	220	45.6
Low education^c^	1280	22.9	373	16.3	221	13.3	397	34.1	289	60.0
Low job control	1563	27.9	97	4.2	226	13.6	818	70.3	422	87.6

^a^6 years of schooling

^b^Score 1–3

^c^≤9 years of schooling

In bivariate analyses, all covariates (paternal education, IQ, educational level and job control) were associated with overall DP (Table 4). Among men, lower educational levels displayed the strongest associations. Also, a clear gradient was shown for the association between levels of education and DP among men, while among women, only the two lowest educational levels displayed increased risk of DP. For women, low job control showed the strongest association.

**Table 4:** Bivariate associations between covariates and disability pension due to all causes in unadjusted hazard ratios (HR) with 95 % confidence intervals (CI)

	Men		Women	
	HR^a^	95 % CI	HR^a^	95 % CI
Paternal education				
Not low	1		1	
Low	1.37	1.09–1.72	1.33	1.12–1.58
IQ				
Low (1–3)	1.52	1.27–1.81	1.58	1.37–1.82
Average (4–6)	1		1	
High (7–9)	0.59	0.47–0.74	0.94	0.80–1.11
Education				
≤9 years	4.05	2.91–5.63	1.87	1.53 -2.28
10–11 years	3.19	2.28–4.48	1.29	1.06–1.57
12–13 years	2.51	1.74–3.63	0.82	0.60–1.10
14 years	1.84	1.18–2.85	0.96	0.76–1.23
≥15 years	1		1	
Job control				
High	1		1	
Medium–high	1.57	1.26–1.95	1.62	1.22–2.17
Medium–low	2.04	1.63–2.57	1.50	1.13–1.98
Low	2.19	1.77–2.72	2.41	1.82–3.18

^a^Adjusted for birth cohort

High and medium–high physical workload were, compared to low load, associated with higher HR of overall DP, DP due to musculoskeletal disorders and others causes among both men and women (Tables 5, 6). The crude associations were strongest for DP due to musculoskeletal disorders (HR 5.44, 95 % CI 3.35–8.84 for men with high load, HR 3.82, CI 95 % 2.88–5.08 for women with high load). There were no associations between physical workload and DP due to psychiatric disorders, except for a reverse association seen for men in the fully adjusted model. In multivariate analyses, all associations were clearly attenuated after adjustments for early life factors, educational level, and simultaneous exposure to job control. For the association between physical load and DP due to musculoskeletal disorders, slightly larger attenuations were shown after adjustments for education and job control compared to adjustments for the early factors. Nevertheless, when adjustments had been made for all potential confounders a clear association between high physical workload and DP due to musculoskeletal disorders remained for both men and women (HR 2.25, 95 % CI 1.28–3.94 for men, HR 2.19, CI 95 % 1.48–3.23 for women). For women also the slightly weaker associations between medium–high load and musculoskeletal DP, as well as between both high and medium–high physical workload and overall DP, remained significantly increased.

**Table 5:** Association between physical workload 1985–1990 and disability pension (DP) due to all causes, musculoskeletal disorders, psychiatric disorders and others causes 1991–2009 among men, as hazard ratios (HR) with 95 % confidence intervals (CI)

Physical workload	Low	Medium–low		Medium–high		High	
		HR^a^	95 % CI	HR^a^	95 % CI	HR^a^	95 % CI
*DP: total (n* = *620)*							
Crude	1	1.15	0.89–1.48	2.14	1.66–2.76	2.07	1.64–2.62
Adjusted for							
Paternal education	1	1.13	0.88–1.46	2.05	1.58–2.66	1.98	1.56–2.52
IQ	1	1.16	0.90–1.50	1.80	1.39–2.34	1.74	1.36–2.21
Education	1	1.12	0.87–1.45	1.52	1.16–1.98	1.43	1.11–1.85
Job control	1	1.05	0.81–1.37	1.63	1.20–2.20	1.57	1.19–2.08
All covariates	1	1.08	0.84–1.41	1.21	0.89–1.65	1.13	0.84–1.51
*DP: musculoskeletal (n* = *224)*							
Crude	1	1.60	0.92–2.78	3.87	2.29–6.55	5.44	3.35–8.84
Adjusted for							
Paternal education	1	1.58	0.91–2.74	3.62	2.12–6.16	5.08	3.10–8.34
IQ	1	1.61	0.93–2.79	2.97	1.74–5.06	4.15	2.54–6.81
Education	1	1.56	0.89–2.71	2.41	1.40–4.15	3.30	1.99–5.48
Job control	1	1.39	0.80–2.44	2.64	1.45–4.81	3.62	2.07–6.32
All covariates	1	1.48	0.85–2.57	1.68	0.92–3.08	2.25	1.28–3.94
*DP: psychiatric (n* = *130)*							
Crude	1	0.82	0.52–1.31	1.16	0.71–1.91	0.76	0.47–1.24
Adjusted for:							
Paternal education	1	0.82	0.51–1.31	1.14	0.69–1.88	0.75	0.45–1.23
IQ	1	0.83	0.52–1.32	1.08	0.66–1.79	0.70	0.42–1.16
Education	1	0.81	0.51–1.29	0.88	0.52–1.49	0.57	0.34–0.96
Job control	1	0.74	0.46–1.19	0.70	0.38–1.30	0.51	0.29–0.91
All covariates	1	0.77	0.48–1.23	0.58	0.30–1.09	0.40	0.22–0.74
*DP: other causes (n* = *266)*							
Crude	1	1.21	0.82–1.76	2.16	1.48–3.15	1.67	1.16–2.40
Adjusted for:							
Paternal education	1	1.19	0.81–1.73	2.09	1.42–3.06	1.60	1.10–2.32
IQ	1	1.23	0.85–1.80	1.87	1.27–2.74	1.44	0.99–2.09
Education	1	1.18	0.81–1.72	1.60	1.07–2.39	1.21	0.82–1.79
Job control	1	1.16	0.79–1.70	1.95	1.25–3.04	1.46	0.95–2.24
All covariates	1	1.18	0.80–1.72	1.52	0.95–2.41	1.09	0.70–1.71

^a^Adjusted for birth cohort

**Table 6:** Association between physical workload 1985–1990 and disability pension (DP) due to all causes, musculoskeletal disorders, psychiatric disorders and others causes 1991–2009 among women, as hazard ratios (HR) with 95 % confidence intervals (CI)

Physical workload	Low	Medium–low		Medium–high		High	
		HR^a^	95 % CI	HR^a^	95 % CI	HR^a^	95 % CI
*DP: total (n* = *1006)*							
Crude	1	1.09	0.92–1.28	1.64	1.39–1.92	2.33	1.92–2.82
Adjusted for:							
Paternal education	1	1.08	0.92–1.27	1.57	1.33–1.84	2.20	1.81–2.67
IQ	1	1.07	0.91–1.26	1.50	1.27–1.78	2.02	1.65–2.48
Education	1	1.13	0.94–1.36	1.48	1.24–1.76	1.93	1.56–2.38
Job control	1	1.08	0.91–1.27	1.48	1.20–1.81	2.05	1.60–2.63
All covariates	1	1.11	0.92–1.34	1.34	1.08–1.66	1.64	1.26–2.14
*DP: musculoskeletal (n* = *443)*							
Crude	1	1.30	1.00–1.70	2.45	1.91–3.15	3.82	2.88–5.08
Adjusted for							
Paternal education	1	1.29	0.99–1.68	2.27	1.76–2.92	3.48	2.61–4.63
IQ	1	1.25	0.96–1.64	2.12	1.64–2.74	3.13	2.33–4.23
Education	1	1.40	1.04–1.87	1.96	1.50–2.55	2.74	2.02–3.72
Job control	1	1.31	1.00–1.73	1.96	1.42–2.68	2.96	2.05–4.29
All covariates	1	1.42	1.04–1.94	1.63	1.18–2.25	2.19	1.48–3.23
*DP: psychiatric (n* = *247)*							
Crude	1	0.99	0.74–1.34	0.96	0.68–1.36	1.21	0.77–1.90
Adjusted for							
Paternal education	1	0.99	0.73–1.34	0.94	0.67–1.34	1.17	0.74–1.84
IQ	1	1.00	0.74–1.35	0.97	0.68–1.39	1.19	0.74–1.91
Education	1	1.05	0.74–1.47	0.99	0.68–1.43	1.13	0.70–1.84
Job control	1	0.98	0.72–1.34	0.95	0.62–1.46	1.17	0.68–2.03
All covariates	1	1.08	0.75–1.54	1.02	0.65–1.60	1.11	0.62–1.99
*DP: other causes (n* = *326)*							
Crude	1	0.96	0.72–1.27	1.45	1.09–1.92	1.86	1.31–2.64
Adjusted for							
Paternal education	1	0.96	0.72–1.27	1.41	1.06–1.87	1.80	1.26–2.57
IQ	1	0.94	0.71–1.25	1.33	0.99–1.78	1.57	1.08–2.28
Education	1	0.94	0.68–1.30	1.36	1.01–1.85	1.66	1.14–2.43
Job control	1	0.94	0.70–1.25	1.48	1.03–2.10	1.87	1.20–2.92
All covariates	1	0.89	0.64–1.24	1.31	0.90–1.90	1.46	0.91–2.34

^a^Adjusted for birth cohort

## Discussion

This is one of the very few studies, and among the largest, on the association between high physical workload and disability pension that includes both men and women and where information on workload is not self-reported, but based on a measure attributed from occupational titles. Unlike most previous studies on physical workload, the associations have been adjusted for a panorama of risk factors; from early life factors to coincident psychosocial working conditions. The study has applied a life course perspective by adjusting for possible confounding by early life factors that may have acted as selective forces into working life. The study shows that long-term exposure to high physical workload measured 5 years apart at around age 40 was strongly associated with disability pension due to musculoskeletal disorders up to the age of 61 among both men and women. For women, the association between high workload and overall DP was also significantly increased. The increased risks remained, but were clearly attenuated after adjustment for fathers’ education, IQ in childhood, achieved education, and concomitant level of control at work. High physical load was not associated with higher incidence of DP due to psychiatric disorders. For men, high physical load was even associated with a decreased risk of DP due to psychiatric disorders.

### Comparison with previous studies

The results are in accordance with several earlier studies showing that high physical load may increase the risk of DP (Friis et al. 2008; Jarvholm et al. 2014; Karkkainen et al. 2013; Karpansalo et al. 2002; Labriola et al. 2009b; Lahelma et al. 2012). Those studies that have investigated risks of DP in specific diagnosis groups have, similar to our findings, found that the risk is increased for DP due to musculoskeletal disorders, but not due to mental disorders (Karpansalo et al. 2002; Lahelma et al. 2012). Even in a cohort consisting of mainly manual workers, i.e. a cohort of Swedish construction workers, the risk of DP varied considerably with the largest risks found for the occupations with the heaviest work tasks, for example rock workers and roofers (Jarvholm et al. 2014).

A few other studies on the association between high physical workload and DP have controlled for possible confounding by psychosocial working conditions and have indicated independent effects (Friis et al. 2008; Krokstad et al. 2002; Labriola et al. 2009b; Lahelma et al. 2012). A Finnish study (Lahelma et al. 2012) found that high physical load and low job control were the primary risk factors for all-cause DP and DP due to musculoskeletal disorders, after adjustment for a wide range of other working conditions. In our study, job control explained more of the association between physical workload and DP due to musculoskeletal disorders than the early factors, and to roughly the same extent as educational level.

Our results are also consistent with a few studies that have adjusted the association between high physical workload and DP for educational level and found remaining effects (Karpansalo et al. 2002; Krokstad et al. 2002; Lahelma et al. 2012). However, most of the prior studies have not controlled for potential confounding due to early life factors. A Finnish twin study found that several adverse physical and psychosocial work characteristics were strongly associated with DP due to musculoskeletal diagnoses, even after adjustments for family background, including genetics, and shared environment (Karkkainen et al. 2013).

A few studies of the association between educational level and DP have controlled for risk factors established in childhood and adolescence. In a Swedish conscription cohort, a large part of the association between level of education and DP in middle age was explained by factors measured in late adolescence, and by IQ in particular (Johansson et al. 2012). Falkstedt et al. (2014) found that factors present in late childhood, such as SEP and IQ, contributed to the association between education and DP.

Robust associations between low IQ in adolescence and DP have been shown in Swedish conscription cohorts; both with DP in early adulthood (Upmark et al. 2001) and in middle age (Sorberg et al. 2013). The associations were found to be attenuated, but still significant, after adjustments for factors such as social background, education, SEP, and working conditions. Also in a recent study of DP in cause-specific diagnoses, associations between intelligence in childhood and DP overall and DP due to musculoskeletal disorders were partly mediated through educational level, physical and psychosocial working conditions, indicating that some of the associations could be explained by a selection of groups with lower intelligence into jobs with poor working conditions (Lundin et al. 2015). These associations were stronger than the associations between intelligence and DP due to mental disorders and cardiovascular disease. However, these studies indicate that the association between intelligence and DP is not fully mediated through education and working conditions. Intelligence has been shown to explain variations in health behaviors such as smoking, diet habits, and physical activity (Deary et al. 2010). It is also reasonable to believe that intelligence influences risk behaviors in working life. A higher intelligence might imply a better ability to manage high physical loads in working life, for example by using appropriate working techniques that reduce loads on the musculoskeletal system, and to understand and adhere to safety regulations.

### Interpretation of findings

A DP decision by the Swedish Social Insurance Agency is based on an assessment of whether the individual’s work ability is permanently reduced (SFS 2010). Work ability, in turn, is determined both by the individual’s health and/or capacity and the working conditions. In this study, the focus was on physical working conditions as a risk factor for DP, while controlling for psychosocial conditions.

The main finding was that of high physical load being a strong and independent risk factor for DP due to musculoskeletal disorders. We adjusted for a panorama of risk factors, from early life factors to coincident psychosocial working conditions, which may possibly involve some overadjustment. As discussed earlier, intelligence and childhood SEP select into education and occupation, but have also been suggested as risk factors in themselves for DP. Further, a concern is that physical and psychosocial exposure often coincide in the same occupations (Punnett 2014). Nevertheless, a strong association remained. A likely explanation for this association is that high biomechanical load causes harmful effects to the musculoskeletal system, and along with musculoskeletal injuries and disorders the physical capacity decreases; an explanation of which there is a large amount of evidence for (da Costa and Vieira 2010; Punnett 2014; Punnett and Wegman 2004). The finding that the association between high physical load and DP was restricted to DP in musculoskeletal diagnoses, and not to DP in psychiatric disorders, further supports this effect of high physical load.

An additional explanation is probably that the opportunities to continue to work with reduced health and function are less in heavy manual jobs than in higher skilled non-manual jobs. Low-skilled jobs comprising manual work tasks and high physical load are often characterized by low job control (Kausto et al. 2011; MacDonald et al. 2001; Schrijvers et al. 1998). This co-occurrence of adverse physical and psychosocial working conditions has been explained as a structural feature of how work is organized resulting in a division of labor into mental (task planning) and physical work (task execution) (MacDonald et al. 2001; Punnett 2014). In our material, job control and physical workload were clearly correlated, with the highest proportions of employees with low job control in high and medium–high physical workload jobs. In the Finnish study mentioned earlier (Lahelma et al. 2012), among a wide range of working conditions, low job control, along with high physical load, emerged as a primary risk factor for DP due to musculoskeletal disorders. Apart from the fact that pain and injuries in themselves can be obstacles to work, a hindrance to continue to work in heavy manual jobs may be the high physical demands and low control in such jobs, implying low possibilities to adjust work. In high-skilled jobs, the flexibility is often higher which may make health problems less of a hindrance to continue working (Johansson and Lundberg 2009). The finding by others that the association between educational level and DP (Bruusgaard et al. 2010; Falkstedt et al. 2014), as well as between SEP and DP (Leinonen et al. 2011), is strongest for DP in musculoskeletal diagnoses, provide further support for the assumption that working conditions in low-skilled manual jobs are particularly incompatible with impaired physical health.

Additionally, our analyses indicate that the association between high physical load and DP due to musculoskeletal disorders may to some extent be due to a selection of individuals with lower intelligence and low level of education into heavy physical jobs.

Our results indicate that physical workload does not increase the risk for DP due to psychiatric disorders. This may imply that high physical workload does not constitute an obstacle to work in the presence of mental illness, or expressed in another way, that mental illness does not reduce the ability to work in manual jobs. The negative association between physical workload and DP due to psychiatric disorders for men in the fully adjusted model raises a question of whether high physical work even are protective to mental illness? Another interpretation is that physicians might tend to diagnose individuals with manual occupations more often with musculoskeletal diseases than individuals with nonmanual occupations. It has been shown that a large part of DP in somatic diagnoses can be attributed to common mental disorders (Knudsen et al. 2010; Rai et al. 2012). Also, both of the last-mentioned interpretations are in line with findings from a Swedish study indicating an association between lower level of education and lower risk of DP due to mental diagnoses among women (Samuelsson et al. 2012).

### Methodological considerations

The strengths of the present study include that information on physical workload was based on a measure attributed from occupational titles, and not self-reported, and that long-term exposure was used, based on 5 years of stable exposure. In most studies on high physical workload and DP, the workload have been self-reported, and often from only one occasion in a baseline questionnaire (Friis et al. 2008; Karpansalo et al. 2002; Lahelma et al. 2012). The study is one of the largest to date on the association between high workload and disability pension and includes both men and women. A major strength is also the access to prospectively measured information from age 13 in compulsory school in 1961 and 1966, register-based data on education and occupation in middle age and a long-term follow-up until the ages of 56 and 61 years from national records of DP in different diagnoses. The time for follow-up is longer than in most other studies on physical workload and DP; 19 years after the exposure to physical workload was collected. The population was randomly selected, and the participating rate was very high with more than 90 % participating children. The analytical sample is most likely representative of Swedish workers with a stable exposure over longer periods of time during those years. We did not have data on health or health-related lifestyle factors from childhood. However, when Johansson et al. (2012) examined the extent to which multiple factors from late adolescence could explain the association between level of education and DP, the largest part of the association was explained by differences in terms of IQ scores. Musculoskeletal and psychiatric diagnoses as well as health-related lifestyle factors (overweight, smoking and risky alcohol consumption) were of limited importance. Since our cohorts are from a similar period as Johansson et al. (2012) we have no reason to believe they are different.

Physical workload and job control were estimated with JEMs based on job titles. To use JEMs to estimate exposure in work has been proposed as a more objective way than to use self-reported exposure, as the information is not influenced by individuals’ claims, expectations and health, and thus the risk of differential misclassification of exposure is eliminated (Fredlund et al. 2000). However, to use a JEM to obtain exposure information also has limitations. The true variations in exposure between individuals within the same occupation are eliminated, which may lead to underestimation of true associations. However, in two different studies, comparisons have been made of risk estimates of coronary heart disease obtained when exposure to job control has been inferred from job titles and when self-reported job control has been used, and similar associations have been found (Bosma et al. 1997; Theorell et al. 1998). These JEMs have been used in previous studies showing associations between physical workload as well as psychosocial working conditions and DP (Falkstedt et al. 2014; Samuelsson et al. 2013). In the psychosocial JEM, also a measure of psychological demands at work was available. That measure, as well as previous job demand JEMs, has been found to correlate only weakly with self-reported measures of job demand (Fredlund et al. 2000; Theorell et al. 1998), and therefore we decided not to use this measure in our study.

In order to obtain a measure of long-term exposure to physical load, we restricted the data set to those stable in one of the four groups of physical workload (low, medium–low, medium–high, and high) between the years 1985 and 1990. There are reasons to believe that there may partly be a selection of fit and healthy workers that stay in heavy jobs, which can lead to underestimation of the effect of high physical workload on DP (Östlin 1989a).

## Conclusions

Our study shows that long-term exposure to high physical workload is strongly associated with risk of DP due to musculoskeletal disorders among both men and women, and also with the overall DP among women, even though adjustments for childhood SEP, IQ in childhood, educational level and job control clearly attenuated the risks.

Reasons for not being able to continue to work in jobs involving high physical load may partly be that the high load causes musculoskeletal disorders and injuries, along with reduced physical capacity, and partly that the opportunities to continue to work with a reduced health and function are particularly small in these jobs due to high physical demands and low possibilities to adjust work. Measures to prevent early exits from the labor market through disability pensions in musculoskeletal diagnoses should therefore include not only elimination of hazardous physical load in working life, but also promotion of working conditions that allows for adjustments of work to a reduced physical capacity.
